# The Influence of Discrepancies between Parents’ Educational Aspirations and Children’s Educational Expectations on Depressive Symptoms of Left-Behind Children in Rural China: The Mediating Role of Self-Efficacy

**DOI:** 10.3390/ijerph182111713

**Published:** 2021-11-08

**Authors:** Xiaoou Man, Jiatong Liu, Yutong Bai

**Affiliations:** 1 School of Humanities and Law, Northeastern University, No. 195 Chuangxin Road, Hunnan District, Shenyang 110169, China; jiatong_liu@outlook.com; 2 School of Public Administration, Inner Mongolia University of Finance and Economics, No. 185 North 2nd Ring Road, Huhhot 010070, China; Bai_Yutong@outlook.com

**Keywords:** aspiration-expectation discrepancy, left-behind children, depressive symptoms, academic self-efficacy

## Abstract

Although long-term separation has made discrepancies between parents’ educational aspirations and children’s own educational expectations among families with left-behind children (LBC), limited researches on the influence of these discrepancies on children’s mental health are carried out at present. Based on China Family Panel Studies (CFPS) conducted in 2018, we selected 875 LBC aged 9~15 as the sample, explored the influence of the direction and degree of these discrepancies on LBC’s depressive symptoms by hierarchical regression, and examined the mediating role of children’s academic self-efficacy and mediation effect pathway with Baron and Kenny method and Bootstrap mediation analysis methods. Results showed that LBC’s mental health was worse when parents’ educational aspirations were higher than their children’s educational expectations, compared to that without discrepancies. The degree of such discrepancies was negatively associated with LBC’s mental health. In the relationship between the direction of discrepancies and LBC’s depressive symptoms, academic self-efficacy played a mediating role partially. In addition, the study indicated that mothers played a significant role in the development of LBC’s mental health. These findings also provided critical evidence for the intervention practice of LBC’s mental health.

## 1. Introduction

China’s rapid urbanization has caused large numbers of rural residents migrating to urban areas for work, resulting in the emergence of a potentially vulnerable sub-population, left-behind children (LBC) [1,2]. LBC refers to children who are under 18 with one or both parents migrating to cities for work for at least 6 months, while children remain rural household [3,4]. They are usually taken care of by one parent, grandparents, siblings, other relatives or friends, etc. [5]. A large number of studies have shown that long-term separation from parents would arise multiple detrimental impacts on LBC, for example: prosocial behavior [6], obsessive-compulsive [7], depression [8,9], lower academic performance [10], emotional symptoms [6,11], and peer problems [11].

Researches on the mental health of left-behind children indicated that factors such as children’s age [12], gender [13], personality and self-esteem [7], self-efficacy [14], types of family migration [15], caregivers’ individual factors (such as age, educational attainment, socioeconomic status, etc.) [9,11], duration of separation [16,17], communication duration and frequency [18], as well as coping strategies in dealing with daily stressors [19] all had significant influence. In addition, some studies analyzed the effect of parents’ migration on their left-behind children’s academic performance. Lu [15] argued that parents’ migration did not mean that LBC achieved performance as their parents’ hope. To provide better financial support for their children, parents migrate to urban areas and place greater aspirations on their children. Their attitudes towards children’s education gradually shift from the idea of “uselessness of education” to “no matter how poor you are, you cannot be poor in education”. However, the long-term separation makes parents know little about their children’s actual academic performance, so parents’ aspirations of “hoping their children to have a bright future” are often different from LBC’s own educational expectations. 

As for educational aspiration and educational expectation, existing researches distinguish the difference between the two concepts: educational aspiration refers to one’s hope or goal on future educational achievements [20], while educational expectation refers to individual expectations on future educational achievements (such as the highest level of education attainment) and realistic beliefs or judgments on future educational achievements [21]. Although both of them target at future achievements, essential difference still exists in the reference on which standards are formed. Taking learning capabilities and realistic resources into account, educational expectation is usually measured by the level of attainments likely to be achieved by asking a person “thinks” one’s educational attainments [21]. Compared to educational expectation, educational aspiration is usually higher and more stable, but less likely to be realized. Based on individual-level aspirations and society consensus concerning education’s benefits, educational aspiration is measured by the level of attainments possibly achieved under ideal conditions, by asking a person “hopes or wishes” one’s educational attainments [21]. Parents’ educational aspiration usually refers to desire, wish or goal formed by themselves on their children’s future achievements, rather than what they expect their children to achieve in reality, which reflects parents’ value judgment on education.

Since educational expectations and education aspirations are expectations or hopes of future educational achievements, researchers mainly take children’s academic performance as the outcome variable when exploring discrepancies on educational expectations or aspirations [22]. For example, Kirk [23] selected 171 families of the United States and found that children were likely to have lower academic performance when parents’ educational expectations exceeded their children’s educational aspirations. Boxer et al. [24] selected 761 students from a public middle school in the United States in order to examine the relationship between students’ aspiration-expectation discrepancies and factors such as academic performance and socioeconomic factors. They found that adolescents whose aspirations exceeded their expectations would achieve lower grades. Based on the dataset of Education Longitudinal Study of 2002 in United States, Trinidad [25] found that students’ school performance was negatively associated with parents’ over-aspiration (higher than children’s educational expectation) with a sample of 7635 students from senior high school students (Grade 10) in the United States. 

However, as we paid more attention to children’s psychological problems in demography, sociology and other disciplines and tried to determine the stressors affecting children’s mental health [26], researches on parent-children educational expectations or aspirations discrepancies also took psychological well-being into consideration. In spite of this, there are still very limited studies focused on the relationship between discrepancies (between parents’ educational aspirations and children’s expectations) and children’s psychological well-being. Wang & Benner [27] chose 14,041 students as samples from National Education Longitudinal Study of 1988. The result indicated that the perceived differences in educational expectations between parents and children caused academical pressure on children, which could be negatively associated with children’s psychological well-being. Based on Waves I and II of the National Longitudinal Study of Adolescent Health, Gallagher and other scholars [28] explored the discrepancies between perceived parents’ educational aspirations and children’s educational aspirations under the premise of increasingly greater significance of higher education. They found that such discrepancy was a pressure source for children, and even a very small difference may also bring in psychological issues. As for the influence of discrepancies between parents’ educational aspirations and children’s educational expectations on children’s psychological well-being, to our knowledge, only one cross-sectional research conducted by Rutherford [29] focused on this question. With a sample of middle school students and high school students throughout the United States, it indicated that middle school students whose expectations matched with their parents’ aspirations were higher on the level of psychological well-being compared with inconsistent group.

Although prior researches discussed above have measured academic performance as outcome variables when exploring educational expectations/aspirations discrepancies, insufficient work has focused on the influence of mental health, or mental health was only considered as an “additional” outcome variable. Given that one’s psychological well-being in childhood is related to that in adulthood [29], we investigated the effect of educational expectations or aspirations discrepancies on children’s mental health. In addition, existing research mostly focused on educational expectations/aspirations discrepancies of the same subjects, but rare researches demonstrated these discrepancies among different subjects, or discrepancies between parents’ idealized “educational aspirations” and children’s actual “educational expectations” of themselves and their influences. Moreover, the effect of this difference on LBC has not been studied yet as far as we know. In terms of research design, previous researches only focused on whether educational aspiration or expectation could match, but little further attention was paid to how the direction (high or low) or the degree (the absolute value of the raw difference scores) of discrepancies exerted impacts. Therefore, this paper aimed to fill up research gaps above by exploring the influence of the direction and degree of discrepancies between parents’ educational aspirations and children’s educational expectations on LBC’s mental health after controlling LBC’s individual and family factors. 

In addition to exploring the direct effect of discrepancies between parents’ educational aspirations and children’s educational expectations on LBC’s psychological well-being, the research aimed to examine the possible mediating variable and its effects. Previous studies have shown that there was a mediating effect of children’s self-efficacy between discrepancies (between parents’ educational aspirations and children’s educational expectations) and children’s academic performance [30]. Self-efficacy is one’s belief that he or she can complete a task or achieve a goal [31,32]. According to social-cognitive theory, people with positive self-efficacy can deal with stress more effectively, improve physical and mental health as well as enhance adaptiveness [33,34,35]. Therefore, this paper would explore whether academic self-efficacy had a mediating effect between discrepancies (between parents’ educational aspirations and children’s educational expectations) and LBC’s depressive symptoms. In summary, we proposed hypotheses as follows:

**Hypothesis** **1** **(H1).** *The direction of discrepancies between parents’ educational aspirations and children’s educational expectations would be positively associated with LBC’s depressive symptoms. In other words, the situation that parents’ educational aspirations are higher or lower than their children’s educational expectations would affect LBC’s depressive symptoms significantly.*

**Hypothesis** **2** **(H2).** *The degree of discrepancies between parents’ educational aspirations and children’s educational expectations would be positively correlated to the depressive symptoms of LBC, namely, the greater the discrepancy between parents’ educational aspirations and children’s educational expectations is, the more depressive LBC are.*

**Hypothesis** **3** **(H3).** *Academic self-efficacy would play a mediating role in the relationship between discrepancies (direction or degree) and LBC’s depressive symptoms.*

## 2. Materials and Methods

### 2.1. Data Sources and Study Design

#### 2.1.1. Data

The research was based on the data from Chinese Family Panel Studies (CFPS) conducted in 2018. CFPS is a nationwide longitudinal social survey conducted by Institute of Social Science Survey in Peking University, using computer-aided survey technology in interviews. In CFPS, participants were conducted by face-to-face interviews aided by computer-assisted personal interviewing (CAPI) technology, computer-assisted telephone interviewing (CATI) technology and computer-assisted Web interviewing (CAWI) technology. It is designed to collect the information of all family members, including family structure, migration status, educational situation, physical and mental health, children’s development, etc. The information covers 25 provinces/autonomous regions, representing 94.5% of the population in mainland China. The sex-age pyramid structure of data is almost in line with that of the census, with nationwide representativeness [36]. 

#### 2.1.2. Sample and Procedure

CFPS collected data from 14,296 households in 2018, divided into databases of children, adults and families. In this study, the sample came from children database aged 9 to 15. 1025 samples of LBC were selected according to “the status of current household registration (rural)” and “duration of living with parent(s) in the past 12 months (less than 6 months)”. Considering the severe impact of parents’ divorce or death on LBC’s psychological well-being, we excluded these two factors to examine the influence of discrepancies between parents’ educational aspirations and children’s educational expectations on LBC’s mental health. After excluding the samples of parents’ divorce, death (130 samples) and invalid ones (20 samples) as well as matching with the database of adults and the database of family according to family ID, the final sample came from 875 groups of LBC aged 9 to 15 and their parents in rural China. The reliability factor Cronbach’s α was 0.8401.

### 2.2. Measures

#### 2.2.1. Dependent Variables

Depressive symptoms. CES-D (The Center Epidemiologic Studies Depression Scale) was used to analyze the mental health level of LBC in rural areas. Developed by Radloff in 1977, CES-D consists of 20 items (16 of which are items with negative emotion, while 4 of which are items with description of positive emotion), which are assessed according to the frequency of occurrence in the past week (1 = less than a day, 2 = sometimes, 3 = often, 4 = most of the time) [37,38]. CFPS 2018 adopted a compact model CES-D8 consisting of 8 items and used a percentile equalization method to generate a comparable score CES-D20sc. The higher score means the more symptoms of depression. A score between 16 and 28 indicates the tendency of depression, while a score above 28 indicates the tendency of severe depression. This study measured the dependent variable “depressive symptoms”, namely CES-D score.

#### 2.2.2. Independent Variables

As for the independent variables, “parents’ educational aspiration” corresponded to “children’s educational level to be hoped” in the questionnaire, and “children’s educational expectation” corresponded to “the least educational level to be achieved” answered by children. In terms of the differences between parents’ educational aspirations and children’s educational expectations, we simultaneously considered the two aspects as below: the first one was the direction of the differences between parents’ educational aspirations and children’s educational expectations; the second one was the degree of the differences between parents’ educational aspirations and children’s educational expectations.

IV1: “The direction of discrepancies between parents’ educational aspirations and children’s educational expectations”. It was divided into three categories: (1) parents’ educational aspirations were higher than children’s educational expectations; (2) parents’ educational aspirations were lower than children’s educational expectations; (3) parents’ educational aspirations were the same as children’s educational expectations.

IV2: “The degree of discrepancies between parents’ educational aspirations and children’s educational expectations” (namely “absolute value of the discrepancies”). We re-coded education levels by years of education: illiterate/semi-literate = 0, elementary school = 6, junior high school = 9, senior high school = 12, college = 15, undergraduate =16, master = 19, doctor = 22. By comparing “parents’ educational aspirations” with “children’s educational expectations”, the absolute value of discrepancies between parents’ educational aspirations and children’s educational expectations was calculated to represent “the degree of discrepancies”.

#### 2.2.3. Mediating Variables: Academic Self-Efficacy

In addition to the direct effect of the direction and the degree of the differences between parents’ educational aspirations and children’s educational expectations on LBC’s depressive symptoms, the mediating effect of the differences between parents’ educational aspirations and children’s educational expectations on LBC’s mental health was also studied. Although Pintrich & Elisabeth (1990) has conducted academic self-efficacy scale [39], limited by questionnaire of CFPS, academic self-efficacy was selected as the mediating variable, which was measured by asking “how would you evaluate your academic performance” in the questionnaires. According to students’ degree of satisfaction, “self-efficacy” was encoded from 1 (“very dissatisfied”) to 5 (“very satisfied”).

#### 2.2.4. Control Variables

Individual-level factors of LBC, including age, gender (male = 0, female = 1), constant communication with parents (yes = 1, no = 0).

Family-level factors of LBC, including migration types of parents (only father’s migration=1, only mother’s migration = 2, both parents’ migration = 3), fathers’ education level, mothers’ education level, annual income of family. 

### 2.3. Statistical Analysis

After controlling the effect of “individual-level factors” and “family-level factors” on LBC, the study first conducted hierarchical regression analysis to examine whether “the direction of discrepancies between parents’ educational aspirations and children’s educational expectations” and “the degree of discrepancies between parents’ educational aspirations and children’s educational expectations” had significant effects on LBC’s mental health level.

Subsequently, the study constructed three regression models to test the mediating effect according to the procedure outlined by Baron and Kenny [40]. First, “the direction of differences” and “the degree of differences” were tested by regression model on the dependent variable “depressive symptoms”; Second, “the direction of differences” and “the degree of differences” were tested by regression model on the mediating variable “academic self-efficacy”; Third, “the direction of differences”, “the degree of differences” and the mediating variable “academic self-efficacy” were tested by regression model on the dependent variable “depressive symptoms”. Bootstrap test was performed afterwards in the study to verify whether there was partial or full mediating effect [41,42]. STATA 16.0 and MPlus 8.3 were used for data analysis.

## 3. Results

### 3.1. Sample Description

As shown in Table 1, the sample included 875 LBC aged 9~15, among whom 467 were male (53.37%) and 408 were female (46.63%). Overall, 60.91% of LBC were left behind by both of their parents and 33.83% were left behind only by their fathers. Over half (53.94%) of LBC had no constant communication with their parents in the past 12 months. On average, the years of schooling of their fathers and mothers were 7.24 years (SD = 3.72) and 5.68 years (SD = 4.10), respectively. The average score of academic self-efficacy was 3.34 (SD = 0.95), between “average” and “satisfied”. 

On average, education years of parents’ aspirations and children’s expectations were 15.48 years (college or above) (SD = 2.35) and 14.31 years (high school or above) (SD = 3.09), respectively. Specifically, 76.2% of parents wished their children to obtain a college degree or above, while only 58.3% of children expected that themselves. In terms of discrepancies between parents’ aspirations and children’s expectations, the average absolute value of discrepancies was 2.27 years (SD = 2.76). More than half of parents’ educational aspirations were the same as their children’s educational expectations (50.46%). 35.85% of parents reported higher educational aspirations than their children’s educational expectations, while 13.69% of parents reported lower aspirations than that of their children. 

As for LBC’s depressive symptoms, the average CES-D score was 30.24 (SD = 6.14). Over half of LBC’s CES-D score (63.20%) exceeded the standard score of 28, suggesting that they were in the tendency of depression.

### 3.2. Group Differences of Depressive Symptoms

Since more than 60% of LBC were considered to be psychologically depressed, the study further analyzed group differences of mental health among LBC with different genders and migration types of parents (see Table 2). The results showed that the CES-D score of male LBC was lower than that of female LBC, indicating that psychological well-being of male LBC was better than that of female LBC, whereas this group difference was not statistically significant. 

The analysis on the depressive symptoms of LBC with different migration types of parents showed that LBC with both parents’ migration got the highest CES-D score, followed by LBC with only mothers’ migration, and LBC with only fathers’ migration got the lowest CES-D score. Among them, there was a significant difference between the CES-D score of LBC with fathers’ migration (M = 29.35, SD = 5.83) and that of LBC with both parents’ migration (M = 30.71, SD = 6.20) (*p* < 0.01), suggesting that children left behind by both parents had the most severe depression compared to children left behind only by fathers.

### 3.3. Hierarchical Regression Analyses Depressive Symptoms

Before testing the hypothesized model, the study examined the correlation as well as the collinearity among all variables. The results manifested that all VIFs were less than 2, without multicollinearity between variables. Hierarchical regression was used to examine the effect of differences between parents’ educational aspirations and children’s educational expectations on LBC’s mental health (see Table 3). We first examined the effect of LBC’s individual-level factors on mental health (Model 1) and the effect of individual factors and family factors on mental health (Model 2). In Model 3, we examined the effect of independent variables after controlling LBC’s individual-and family-level factors on dependent variable. 

Analysis results presented that the direction of the differences between parents’ educational aspirations and children’s educational expectations (parents’ educational aspirations was higher than children’s educational expectations) and the degree exerted significant effects on LBC’s mental health after controlling individual and family factors. Compared with children whose expectations were the same as their parents’ aspirations, depressive symptom level of children whose parents held higher aspirations increased by 1.392 (*p* < 0.05). However, the effect of the direction that “parents’ aspirations were lower than children’s expectations” on LBC’s mental health was not statistically significant, indicating that part of Hypothesis 1 has been supported. IV2 (the degree of discrepancies) was statistically positively associated with LBC’s depressive symptoms (*p* < 0.05), suggesting that when the discrepancies between parents’ educational aspirations and children’s educational expectations increased by every 1 year, the depression tendency of LBC would increase by 28.7% (*p* < 0.05), which verified Hypothesis 2. In addition, compared with children left behind by fathers, psychological well-being of children left behind by both of parents was significantly worse (*p* < 0.01). Moreover, the educational level of mother was found statistically significant in explaining the dependent variable (*p* < 0.001), suggesting that the higher the mother’s educational level was, the better mental health LBC had. Age, sex, communication with parents, father’s education level and family income level did not show significant effects as hypothesized.

### 3.4. Mediating Effect Tests on Depressive Symptoms

**Figure 1:**
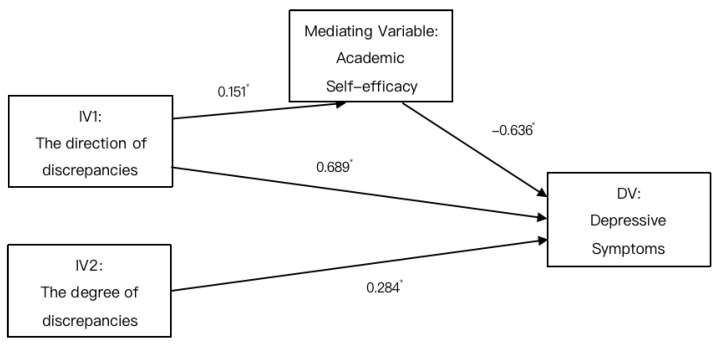
The pathways of LBC’s depressive symptoms. * *p* < 0.05.

The mediating effect was tested by BK method (see Table 4) and standardized Bootstrap method. The results demonstrated that academic self-efficacy (Coeff. = −0.099, *p* < 0.05, CI: −0.163, −0.034) had a partial mediating function on the relationship between IV1 (the direction of discrepancies) and LBC’s depressive symptoms. No mediating effect was found between IV2 (the degree of discrepancies) and LBC’s depressive symptoms. The influencing path of the two dimensions of the differences between parents’ educational aspirations and children’s educational expectations on LBC’s depressive symptoms was shown in Figure 1.

## 4. Discussion

### 4.1. Discrepancies and LBC’s Depressive Symptoms

The research explored the relationship between discrepancies (between parents’ educational aspirations and children’s educational expectations) and mental health of left-behind children in rural China, as well as the mediating effect of children’s academic self-efficacy in this relationship.

Firstly, the proportion of parents who held higher aspirations than their children’s expectations was higher among left-behind children in rural areas compared to general children. Compared with children in CFPS 2018, this proportion was even higher than that in the overall statistics (35.85% vs. 19.62%). Some researches indicated a positive correlation between the socioeconomic status of families and parents’ educational aspirations [43], suggesting that parents in working class or peasant class were more inclined to hold lower educational aspirations as compared with the middle-class parents. However, the increase of income, along with the impact of surroundings, also raise rural parents’ aspirations for their children’s education after migrating to work. They generate the idea of “wishing their children a bright future”, and hoped their children to be more outstanding in their academic performance [44]. They also regard education as “an open sesame” for job application. And this might be the possible reason that the proportion of parents whose educational aspirations are higher than children’s educational expectations is higher among parents with left-behind children in our study.

Secondly, the direction and degree of the discrepancies between parents’ educational aspirations and children’s educational expectations significantly affected LBC’s mental health. On the one hand, after controlling individual and family variables, LBC whose parents held higher aspirations were less healthy psychologically as compared with LBC whose expectations were the same as parents’ aspirations. The results of some previous researches noted that the differences between parents’ educational aspirations and children’s educational expectations were beneficial to children’s school performance in the early stage, especially when parents provided adequate supports and involvement as well as conveyed positive and confident values to their children [27]. However, the long-term discrepancies, especially over-aspirations (higher than children’s expectations) would make children doubt their own educational goals and academic capabilities, affecting their mental health via academic stress [27]. When parents held lower aspirations than their children’s expectations, it implied that children had higher requirements on themselves. This internal motivation, different from external motivation from parents, exerted less influence on their psychological well-being [25]. Therefore, we found no significant effect of the situation in which parents’ educational aspirations were lower than children’s educational expectations on the mental health of left-behind children.

On the other hand, the larger the discrepancies between parents’ educational aspirations and children’s educational expectations were, the less psychologically healthy LBC were. According to Higgins’ self-discrepancy theory, every individual has three selves: the actual self, the ideal self and the ought-to self [45]. Each self has two perspectives, the person considered by oneself and the person perceived by important others [42]. Parents’ educational aspirations can be regarded as “the ideal person perceived from important others”, while the educational expectations of one’s own can be regarded as “the actual self from oneself”. The differences between parents’ educational aspirations and children’s expectations are that between the ideal self and the actual self. Higgins also found the conflict between the ideal self and the actual self, even a subtle one, would cause psychological issues [45]. Therefore, the greater discrepancies between parents’ educational aspirations and children’s educational expectations meant the larger gap between the ideal self and the actual self, which would lead to the lower mental health level.

Thirdly, academic self-efficacy played a partial mediating role between IV1 (the direction of differences between parents’ educational aspirations and children’s educational expectations) and LBC’s depressive symptoms. In other words, when parents’ educational aspirations were higher than their children’s educational expectations, LBC held the most negative academic self-efficacy, and then LBC had the lowest mental health level; when parents’ educational aspirations were the same as their children’s educational expectations, LBC held most positive academic self-efficacy, and then LBC had the highest mental health level. The existing work of Spera et al. [46] has demonstrated that parents’ educational aspirations were significantly related with children’s academic performance. The situation that parents’ educational aspirations are higher than their children’s educational expectations convey the message that children’s academic performance is not up to the ideal state considered by their parents, which would result in children’s lack of confidence in their own academic performance [47], namely negative self-efficacy, thus generating emotions such as pressure, lack of self-confidence and depression. When parents’ educational aspirations are the same as their children’s educational expectations, children perceive that their current academic performance has achieved parents’ ideal state, and thus increase confidence in academic performance, which would benefit their mental health. Thus, the direction of discrepancies would influence LBC’s psychological well-being via self-efficacy. According to the self-efficacy theory, one’s behavior or psychology is jointly determined by self-efficacy and educational expectation [30]. Some studies showed that self-efficacy played a moderating role between one’s own educational expectations and psychological well-being [31]. In other words, when one’s own educational expectation is high, self-efficacy is higher, and the mental health level is also higher. However, the degree of discrepancies between parents’ educational aspirations and children’s educational expectations is the relationship between parents’ educational aspirations and children’s educational expectations, which could not directly judge the level of children’s educational expectations or the role of self-efficacy. Therefore, self-efficacy has no mediating effect on the relationship between the degree of discrepancies and psychological well-being. 

Last but not least, this study also indicated the key role of mothers in the development of LBC’s psychological well-being. Both the results of group differences analysis and hierarchical regression analysis demonstrated that LBC with only fathers’ migration, namely looked after by their mothers, were the most psychologically healthy. In hierarchical regression analysis, the control variable mothers’ education level significantly affected the mental health of LBC, which was consistent with findings of previous researches that mothers played an essential role in the growth of children [48,49]. Specifically, mothers’ education attainment and education behaviors not only influenced children’s academic performance, but also their psychological well-being.

### 4.2. Limitations and Recommendations for Further Research

Limited by the questionnaires of CFPS 2018, the study had some limitations. Firstly, control variables were set from individual- and family-level according to the characteristics of questionnaires to examine effects of discrepancies between parents’ educational aspirations and children’s educational expectations on LBC’s mental health level. However, the mental health of left-behind children in rural areas is also affected by many other factors, such as parents’ divorce or death [50], school climate [12,51], network addiction [52], peers’ effects [53], etc. Future researches should take these variables into consideration. Secondly, using cross-sectional data from CFPS 2018, the study may exist sample selection bias and potential endogeneity issues. Further researches could weaken these issues by using instrumental variables with Two Stage Least Square method or a Propensity Score-Matching analysis. Furthermore, all assessments were conducted using self-reported data, with all the limitations that these instruments have in regard to the validity of the construct we want to assess. Finally, the sample only represented left-behind children in rural China. Comparative researches should explore the similarities and differences in the influence of the gap between parents’ educational aspirations and children’s expectations on children’s mental health among different countries.

## 5. Conclusions

Despite these limitations, the study extended the specific dimensions of the differences between parents’ educational aspirations and children’s educational expectations (the direction and degree), and expanded the existing studies from the following two aspects: First, from the perspectives of educational aspirations and educational expectations, it emphasized the influence of differences in educational aspirations and educational expectations between different subjects on LBC’s mental health. Second, previous researches have examined the relationship between children’s educational aspirations/expectations and academic self-efficacy or the relationship between academic self-efficacy and children’s depressive symptoms. Our findings demonstrated the pathway among educational aspirations/expectations, academic self-efficacy and mental health, that is, the direction of differences between parents’ educational aspirations and children’s educational expectations exerted an influence on LBC’s self-efficacy, and then affected LBC’s psychological well-being. 

Furthermore, this study provided critical evidence for the intervention practice of LBC’s mental health. First, open communication between parents and LBC is very necessary. Parents should communicate with their children as much as possible to know children’s actual academic performance and provide them with more information about employment, to narrow the aspirations-expectations gap and relieve LBC’s psychological pressure. Second, educators should cultivate LBC’s communication ability and problem-solving ability to improve their self-efficacy and self-confidence, so as to improve LBC’s internal motivation. Finally, more measures should be taken to support LBC’s psychological well-being. Professional psychological counseling, family training and learning guidance are suggested for LBC and their surrogate caregivers in rural China. 

## Figures and Tables

**Table 1:** Descriptive statistics of the sample (*N* = 875).

Variables	Mean	SD	Number of Cases	Total Percent (%)
Depressive symptoms	30.24	6.14		
not depressed			322	36.80%
depressed			553	63.20%
Discrepancy direction	2.13	0.92		
higher			320	35.85%
lower			120	13.69%
the same			435	50.46%
Discrepancy degree	2.27	2.76		
Age	12.54	1.71		
Gender				
male			467	53.37%
Female			408	46.63%
Constant communication with parents	0.46	0.50		
no			472	53.94%
yes			403	46.06%
Migration types of parents	2.27	0.94		
only fathers migration			296	33.83%
only mothers migration			46	5.26%
both parents migration			533	60.91%
Parents’ education level	6.46	3.34		
fathers’ education level	7.24	3.72		
mothers’ education level	5.68	4.10		
Annual income of family	10.79	0.96		
Academic self-efficacy	3.34	0.95		

**Table 2:** Group differences of depressive symptoms within LBC.

Subgroup	N	M(SD)	Mean Differences
Male	467	30.11 (5.86)	−0.281
Female	408	30.39 (6.45)	−0.281
Migration types of parents			
Only fathers migration	296	29.35 (5.83)	
Only mothers migration	46	30.70 (6.84)	
Both parents migration	533	30.71 (6.20)	
Only fathers migration * only mothers migration			−1.351
Only fathers migration * both parents migration			−1.361 **
Only mothers migration * both parents migration			−0.010

Notes: * *p* < 0.05. ** *p* < 0.01.

**Table 3:** Hierarchical regression results using ordinary least square model.

Variable	Model 1	Model 2	Model 3
Individual-level factors			
Age	0.162 (0.122)	0.560 (0.124)	0.093 (0.125)
Gender (female)	0.309 (0.417)	0.470 (0.415)	0.506 (0.417)
Constant communication with parents (yes)	−0.213 (0.419)	−0.200 (0.415)	−0.230 (0.415)
Family-level factors			
Migration types of parents			
Only fathers migration		−1.218 (0.453) **	−1.165 (0.454) *
Only mothers migration		0.141 (0.937)	0.217 (0.937)
Fathers’ education level		0.117 (0.063)	0.122 (0.063)
Mothers’ education level		−0.224 (0.057) ***	−0.221 (0.057) ***
Annual income of family		−0.022 (0.219)	0.006 (0.220)
Discrepancy direction			
Parents’ educational aspirations are higher than children’s educational expectations			−1.392 (0.763) *
Parents’ educational aspirations are lower than children’s educational expectations			−0.741 (0.831)
Discrepancy degree			0.287 (0.133) *
Constant	28.166 ***	30.487 ***	29.599 ***
N	875	875	875
VIF	1.01	1.11	1.57

Notes: * *p* < 0.05. ** *p* < 0.01. *** *p* < 0.001.

**Table 4:** Mediating effect tests on depressive symptoms.

	Model 1	Model 2	Model 3
	DV: Mental health level	Mediating role: Self-efficacy	DV: Mental health level
Discrepancy direction	0.689 (0.370) *	0.151 (0.057) *	0.785 (0.369) *
Discrepancy degree	0.284 (0.123) *	0.024 (0.019)	0.299 (0.123) *
Academic self-efficacy			−0.636 (0.218) *

Notes: * *p* < 0.05.

## Data Availability

The data are available online at http://www.isss.pku.edu.cn/cfps/ (accessed on 10 October 2021).
